# The therapeutic effect of Taijiquan combined with acupoint pressing on the treatment of anxiety insomnia in college students: A study protocol for a randomized controlled trial

**DOI:** 10.3389/fpsyt.2022.961513

**Published:** 2022-08-10

**Authors:** Jianya Deng, Xinyan Liu, Yiming Wang, Jieyang Fan, Li Yang, Jiamin Duan, Yongfang Yuan, Peishu Lan, Zhuoxuan Shan, Junfeng Xiong, Wenyu Peng, Qingfeng He, Yajie Chen, Xiaoxu Fu

**Affiliations:** ^1^School of Clinical Medicine, Chengdu University of Traditional Chinese Medicine, Chengdu, China; ^2^Acupuncture and Tuina School, Chengdu University of Traditional Chinese Medicine, Chengdu, China; ^3^School of Basic Medical Sciences, Chengdu University of Traditional Chinese Medicine, Chengdu, China; ^4^Hospital of Chengdu University of Traditional Chinese Medicine, Chengdu, China

**Keywords:** Taijiquan, acupoint pressing, anxiety insomnia, college students, RCT (randomized controlled trial)

## Abstract

**Introduction:**

Sleep health is an important part of health and has become a common concern of society. For anxiety insomnia, the commonly used clinical therapies have limitations. Alternative and complementary therapy is gradually rising and showing remarkable effect in clinical practice. This is the first study to evaluate the therapeutic effect of Taijiquan combined with acupoint pressing in the treatment of anxiety insomnia in college students and to compare the difference in intervention before and after sleep, to choose the best treatment time.

**Methods and analysis:**

This is a multicenter, single-blind, randomized controlled trial. A total of 126 eligible subjects who have passed the psychological evaluation and met inclusion criteria by completing a psychometric scale will be randomly divided into treatment group A (treat before sleep), treatment group B (treat after sleep) and control group C (waiting list group) in a ratio of 1:1:1. All the three groups will receive regular psychological counseling during the trial, and the treatment groups will practice 24-style Taijiquan and do meridian acupuncture at Baihui (DU20), Shenting (DU24), Yintang (EX-HN3), Shenmen (HT7) and Sanyinjiao (SP6). This RCT includes a 2-week baseline period, a 12-week intervention period, and a 12-week follow-up period. The main results will be measured by changes in the Pittsburgh sleep quality index (PSQI) and Hamilton anxiety scale (HAMA). The secondary results will be measured by the generalized anxiety scale (GAD-7) and insomnia severity index (ISI). The safety of the intervention will be evaluated at each assessment. The statistical analysis of data will be carried out by SPSSV.26.0 software.

**Discussion:**

We expect this trial to explore the effectiveness of Taijiquan combined with acupoint pressing in the treatment of anxiety insomnia in college students and choose the best treatment time by comparison.

**Clinical trial registration:**

[www.ClinicalTrials.gov], identifier [ChiCTR2200057003].

## Background

Anxiety insomnia is one of the most common clinical insomnia types of contemporary young college students. It is a mood disorder with a high incidence of relapse and a long course of the disease (1). College students are often affected by many external pressure factors such as academic performance, interpersonal communication, and social employment, resulting in bad emotions such as anxiety and irritability. If there is no timely effective intervention, they will begin to have symptoms of waking up repeatedly or severe difficulty in falling asleep and get progressively worse. If things go on like this, it will develop into anxiety insomnia (2). Relevant investigation results show that 18.3∼36.6% of college students have sleep quality problems or even sleep disorders, which seriously affect the quality of their study and life (3). Sleep health is an important part of physical and mental health and has become a common concern in society.

Modern studies show that Taijiquan can properly improve the system functions of the body, regulate emotions and relieve fatigue, and can improve the sleep quality of practitioners when the intensity of training is certain (4). The 24-style Taijiquan is selected and used in this study. The 24-style Taijiquan is based on Yang’s Taijiquan and has been compiled. Compared with traditional Taijiquan, it is more refined and standardized, and it fully reflects the characteristics of Taijiquan movement and meets the standard of harmony and unity (5). In the view of Traditional Chinese Medicine, insomnia is mainly caused by the disharmony of Yin and Yang, the imbalance of Ying and Wei, and the disorder of Zang-Fu viscera. The 24-style Taijiquan is influenced by traditional philosophy and contains the theory of balance of yin and yang, reflecting the Chinese culture’s philosophy of the unity of heaven and man and the Tao of nature, which has the effect of harmonizing yin and yang and unblocking meridians, while regulating the idea of balance and exercising the brain’s perception and control precision of the body (6–8). Acupoint pressing is a therapeutic method with meridians and acupoints as the core, combined with massage manipulation. Using the theory of meridians and acupoints to treat insomnia has a long history and its advantages (9). Clinical research results show that Baihui (DU20), Shenting (DU24), Yintang (EX-HN3), Shenmen (HT7), and Sanyinjiao (SP6) are common acupoints for insomnia treatment (10, 11).

For anxiety insomnia, oral drugs and psychological counseling are mostly used in clinical treatment at present. But the drugs have side effects such as addiction and drug resistance, while psychological counseling also has certain limitations. The therapeutic effect of this kind of disease still needs to be improved in clinical research. With the improvement of the modern medical levels, alternative and complementary therapy is gradually rising and showing remarkable effect in clinical practice. The main purpose of this RCT is to evaluate the clinical therapeutic effect of Taijiquan combined with acupoint pressing for college students with anxiety insomnia and to optimize the best treatment time. The results of the study will also provide evidence for the therapeutic effect of traditional exercise therapy and external acupoint therapy in treating anxiety insomnia.

## Methods

### Study design

**FIGURE 1:**
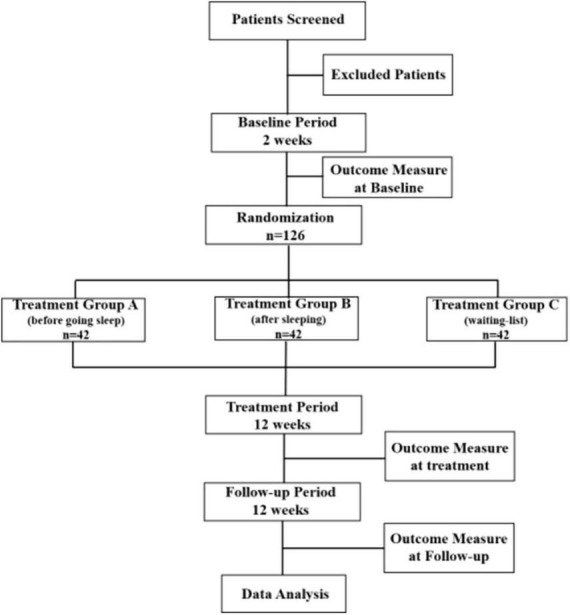
Flowchart of the study design.

**FIGURE 2:**
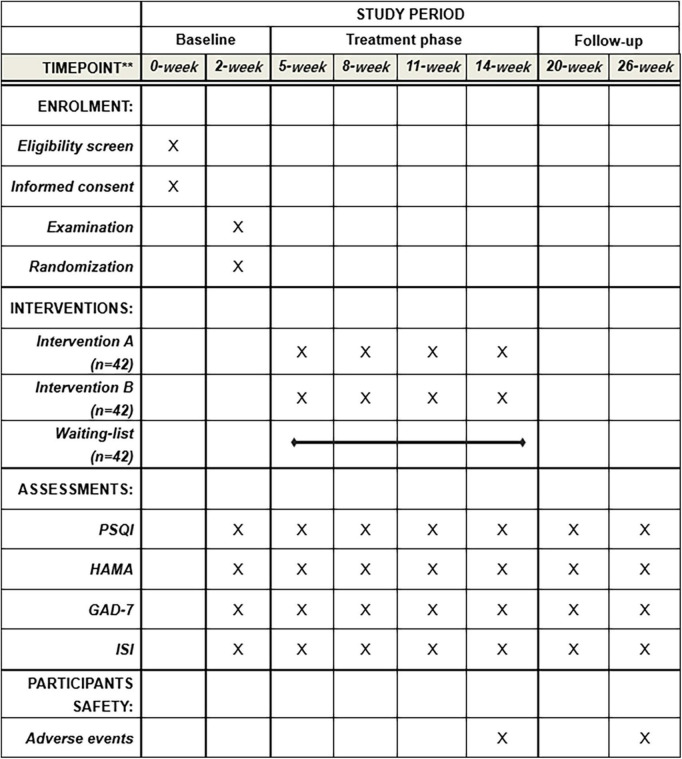
Standard protocol items. PSQI, Pittsburgh sleep quality index; HAMA, Hamilton Anxiety Scale; GAD-7, Generalized Anxiety Disorder; ISI, Insomnia Severity Index.

This proposed study is a multi-centered, single-blinded, randomized non-pharmacological therapy trial with the assessor and statistician blinded to treatment allocation, evaluating the effect of Taijiquan combined with acupoint pressing in the treatment of college students with anxiety insomnia, and finding the best treatment time (12). The research team will include patients with anxiety insomnia according to the international diagnostic criteria DSM-5 (13). A total of 126 participants who have passed the professional psychological assessment will be enrolled in this trial from the universities in Chengdu, Sichuan Province. They will be randomly divided into treatment group A (treat before sleep), treatment group B (treat after sleep), and control group C (waiting list group) in the ratio of 1:1:1. The whole treatment process will be completed under the guidance of professional doctors and teachers of Chengdu University of Traditional Chinese Medicine in Sichuan, China. This test will be reported following the Comprehensive reporting Test Standard (CONSORT) statement (14). The flow chart of the trial is shown in Figure 1, and the schedule for registration, intervention, and evaluation is shown in Figure 2.

### Participants recruitment

There will be three strategies for the recruitment of subjects in this trial. First, participants will be recruited from Chengdu University of Traditional Chinese Medicine. The second is to recruit on the campuses of Sichuan University, University of Electronic Science and Technology, Southwest University of Finance and Economics, Sichuan Agricultural University, and Southwest Jiaotong University and release recruitment information through offline lectures in the teaching building, WeChat, or other online platforms. Third, the information recruitment will be publicly published on the volunteer research platform of the subjects. During the recruitment process, the members of the research team must first introduce the specific research process such as the research periods, intervention methods, and trial groups to the subjects. The subjects must be randomly assigned and abide by the trial arrangements of each group. Researchers invite eligible subjects to join the study and sign an informed consent form and then conduct a baseline assessment. This trial will include 126 subjects according to the diagnostic criteria.

### Inclusion criteria

(1) Adult college students over the age of 18;
(2) Insomnia is diagnosed according to the guidelines for the diagnosis and treatment of insomnia in Chinese adults, and the Pittsburgh sleep quality index (PSQI) score is ≥ 7;
(3) According to the classification and diagnostic criteria of The Diagnostic and Statistical Manual of Mental Disorders (DSM), there is no severe depression and suicidal tendency, and the score on the Hamilton anxiety scale (HAMA) is ≥ 14;
(4) No treatment-related to insomnia or anxiety symptoms in the last 3 months;
(5) Have certain learning ability, be able to practice 24-style Taijiquan independently, find the designated meridians, and complete acupoint pressing;
(6) Voluntarily participate in the experimental study and sign the informed consent and confidentiality letter.

### Exclusion criteria

(1) Meet the classification and diagnostic criteria of The Diagnostic and Statistical Manual of Mental Disorders (DSM) for severe emotional and mental disorders;
(2) Have a history of severe dependence on psychotropic drugs and sleep drugs;
(3) Have a history of serious diseases of important organs (heart, liver, and kidney) or some sudden acute diseases;
(4) Pregnant and lactating women;
(5) Have more serious bad habits affecting sleep, such as smoking, drinking, and Internet addiction;
(6) Recent major emotional trauma;
(7) Any other circumstances that the researchers believe may make the patient unable to complete, comply with, or unsuitable for this study.

### Sample size

This study uses qualitative data group design sample size estimation formula:


$$n_{1}=n_{2}=\frac{\begin{array}{c} {}[z_{\alpha /2}\sqrt{P\,\left(1-P\right)\,\left(1+C\right)/C}\\ +z_{\beta }\sqrt{P_{1}\,\left(1-P_{1}\right)\,P_{2}\,\left(1-P_{2}\right)/C}]{}^{2} \end{array}}{{\left(P_{1}-P_{2}\right)}^{2}}$$

Among them, Zα/2 is the *Z*-value corresponding to the inspection level, Zβ is the *Z* value corresponding to the second type of error probability, *p* is the combined rate of two samples, and p1 and p2 are the two population rates, respectively, c is the ratio of the two sets of sample content, and the inspection level is specified as α = 0.05, test power 1-β = 0.9, c = 1, using a two-sided test, the clinical effective rate of insomnia treatment is calculated as an index. According to related research reports, p1 is 0.68, p2 is 0.92, and p is 0.80. Substituting the formula can get a total sample size of 114 cases (12). Considering possible withdrawal or loss to follow-up during research interventions, the sample size must be expanded to ensure the progress of the research. Therefore, the sample size is expanded by 10% on this basis, and the total sample size is for at least 126 cases.

### Randomization and blinding

After the participants completed the baseline evaluation, they were randomly divided into treatment group A (treat before sleep), treatment group B (treat after sleep), and control group C (waiting list group) according to the proportion of 1:1:1. The random allocation sequence will be generated and hidden by the PLAN program using the statistical software SAS9.1. The random packet sequence is hidden by opaque and sealed envelopes, and the data statistician will not know the result of the allocation (15). This process ensures the full concealment of randomization and is not affected by the experimenter or subjects. Random assignments will be made after the first visit to participants who provide informed consent and meet the inclusion criteria. If the participant is eligible to be included in the study, the researcher will open the corresponding envelope in front of the participant. Envelopes will not be issued until all subjects have completed the baseline assessment. The subjects could not be blinded because of the unique nature of the intervention, but to ensure the scientific nature of the trial, the research team blinded trial evaluators and data, statistical analysts (16). To avoid misleading data analysts, researchers and subjects will be controlled to communicate with them to ensure that later data results are not affected by the subjective judgment.

### Interventions

The treatment groups will practice 24-style Taijiquan under the guidance of professional teachers. The training time is 15 min each time, once a day, for 12 weeks. The essentials of Taijiquan movements are based on the 24-style simplified Taijiquan compiled by the State Sports Commission (now the State Sports General Administration) in 1956 as the standard. The treatment groups will also carry out acupoint pressing training in the acupuncture and massage laboratory of Chengdu university of traditional Chinese medicine, mainly training Baihui (DU20), Shenting (DU24), Yintang (EX-HN3), Shenmen (HT7), and Sanyinjiao (SP6) acupoint positioning and thumb pressing and kneading techniques (17), while the pressing and kneading are required. Generally, pressing for about 1 min. It is appropriate that the patient feels obvious pain at the acupoints and can bear it. Participants will press the above five acupoints once or twice during the test time, and then change one acupoint for 1–2 min and then change one acupoint for a total of about 15 min. The subjects in the treatment group will receive compound treatment for 12 weeks, once a day for 30 min, including 15 min of 24-style Taijiquan and 15 min of acupoint pressing. To maintain the standard of the operation of the experiment, the subjects will practice for 3 days before the intervention, and after the movement is standardized, they will be trained every 2 weeks in Taiji Avenue and acupuncture and massage laboratory of Chengdu University of Traditional Chinese Medicine to avoid forgetting movements. The research team will assess the subjects on 24-style Taijiquan (a score of 60 or above is considered a pass) and if they fail they will be given additional training until they meet the assessment standard. See Table 1 for details of the scoring scale.

**TABLE 1:** Taijiquan mastery rating scale.

Scoring items	Scoring criteria
Hand shapes, maneuvers, steps, footwork, legwork	2 points for each minor error; 3 points for each significant error; 5 points for each serious error; 6–10 points for multiple errors in one movement.
Smooth and precise force points consistent, rounded and coordinated movements	1–3 marks will be deducted for minor deviations from the requirements, 4–6 marks for significant deviations and 7–10 marks for serious deviations.
Natural, focused awareness appropriate speed and outstanding style	1–3 marks will be deducted for minor deviations from the requirements, 4–6 marks for significant deviations and 7–10 marks for serious deviations.
Forgetting the action	3–5 marks will be deducted for each instance of forgetfulness, depending on the degree.
Out of balance	1 point for each wobble; 2 points for each support; 3 points for successive supports.
Number of movements	3 marks will be deducted for each extra or missing movement.
Direction of movement	One point for each deviation of 45° or more from the prescribed direction.
Repeat the set	If the examination is interrupted due to forgetfulness or mistakes, the examination may be repealed once and 10 points deducted, and so on.
Unfinished sets	Those who do not complete the routine will not be graded and will be marked as failing.

A total of 100 points will be awarded, with a total score of less than 60 being unqualified.

Group A receives combined treatment 30 min before sleep. First, 24-style Taijiquan training will be performed for 15 min each time, once a day. Then do acupoint pressing at Baihui (DU20), Shenting (DU24), Yintang (EX-HN3), Shenmen (HT7), and Sanyinjiao (SP6), kneading 1∼2 times for 1∼2 min. And then replacing with the next acupoint, each acupoint will be kneaded 1∼2 back and forth, a total of 15 min of continuous pressing. After each treatment, study members should make strict records to ensure the full participation of subjects in the study (18). Group B does the therapy of Taijiquan combined with acupoint pressing 30 min after waking up, and the treatment procedures and methods are the same as those of Group A. Group C will not accept any intervention on Taijiquan or acupoint pressing, and maintain their normal lifestyle. After the study, subjects in this group can choose to participate in the treatment of 24-style Taijiquan training combined with acupoint massage.

### Follow-up

The purpose of follow-up is to evaluate the long-term effect of Taijiquan combined with acupoint pressing. After completing the treatment, a follow-up evaluation will be performed on the 15th, 18th, 21st, and 24th weeks. Participants will not accept any intervention but only complete the same scales as the trial period during the follow-up period. To encourage participants to comply with the rules, we will give special care to each participant and pay close attention to the changes in their sleep quality.

### Outcome measures

#### Primary outcome measurement

PSQI (19) assesses the sleep quality of the subjects, and HAMA (20) assesses the anxiety severity of the subjects and is jointly used to evaluate the effect of combined treatment. The evaluation time points are in the baseline period (week 2), the trial period (weeks 3, 6, 9, and 12), and the follow-up period (weeks 6 and 12).

#### Secondary outcome measurements

ISI (21) evaluates the severity of subjective insomnia for 2 weeks, GAD-7 (22) can evaluate the degree of anxiety symptoms within 2 weeks and is jointly used to evaluate the degree change of anxiety insomnia. The evaluation time points are the same as the primary outcome index.

#### Safety evaluation

In the process of this research, researchers should monitor and record the adverse events or emotional reactions of subjects, and analyze the causal relationship between the combination treatment and the severity of the adverse events in time (23). During the trial, any adverse events (defined as falls, sprains, any sports-related injuries, etc.) will be recorded. If an adverse event occurs, and the subject has emotions such as mania, severe depression, extreme depression, etc. The experiment should be terminated in time, and psychological counseling or medication should be immediately accepted.

#### Quality control and data collection and management

All researchers will receive a series of training before the start of this study, to ensure that they fully understand the study protocol and standard procedures. CRFs labeled by unique numeric identifiers will be used to collect relevant data, adverse events, and safety assessments for participants. Only outcome assessors have access to CRFs and will perform the double data entry (24, 25). The Evidence-based Medicine Center of the Chengdu University of TCM will be in charge of supervising the study and monitoring data every 3 weeks.

#### Statistical analysis

All data analysis will be conducted by the basic principles of Intentional Analysis (ITT), including data on any participants who withdrew from RCT during the trial (26). The statistical analysis of the data will be carried out using SPSS26.0 software. The data files will be locked after blind review and determined to be reliable and correct, and the whole process of analysis will be unblinded twice.

The full analysis protocol set will be selected for demographic and other baseline characteristics analysis. To evaluate the therapeutic effect and safety, FAS and PPS will be used at the same time. For the efficacy index, endpoint, and outcome index, the intention analysis will be mainly used for the full analysis set (FAS), and the compliance scheme set (PPS) will be analyzed to compare the results of the two data sets; If not, find the reason. After randomization, statisticians will perform safety data set analysis on subjects who received at least one treatment.

This study is a multi-center clinical trial. CMH method was used for counting data, and analysis of variance and analysis of covariance were used for measurement data.

According to factors such as age, gender, history of insomnia, school, etc., a subgroup analysis will be performed to evaluate the difference in intervention efficacy among different populations.

All statistical analysis will be performed in SPSS 26.0 software, *p* < 0.05 will be considered statistically significant, and statistical analysis and supervision will be carried out by statistical experts who do not understand the experimental scheme and group assignment.

#### Patient and public involvement

Patients or the public were not involved in the design, conduct, reporting, or dissemination plans of this research.

#### Ethics and dissemination

This research project was examined and approved by the Ethics Review Committee of the Hospital of Chengdu University of Traditional Chinese Medicine in January 2022 (approval number: 2022KL-004). Before the pilot study, all participants will sign a written informed consent form. The results will be disseminated through peer-reviewed publications or conference reports. The data will be anonymous before publication to prevent the identification of individual participants.

## Discussion

This trial is a randomized controlled clinical trial, which aims to evaluate the efficacy and safety of Taijiquan combined with acupoint pressing in the treatment of anxiety insomnia in college students and to determine the best intervention time for Taijiquan combined with acupoint pressing in the treatment of anxiety insomnia.

Studies have shown that practicing Taijiquan and acupoint pressing can reduce the symptoms of anxiety insomnia to some extent, but the effectiveness of the combination of Taijiquan and acupoint pressing in the treatment of anxiety insomnia is not clear (27, 28). This experiment will be conducted in multiple centers, using a randomized controlled trial to blind the trial evaluators and data statistical analysts to evaluate the effectiveness of Taijiquan combined with acupoint pressing in the treatment of anxiety insomnia in college students. By comparing different treatment times of Taijiquan and acupoint pressing, we can find the best time for the treatment of anxiety insomnia. In addition, this study designed according to the Comprehensive reporting Test Standard (CONSORT) statement and guidelines will provide effective evidence for guiding the clinical practice of Taijiquan combined with acupoint pressing in the treatment of anxiety insomnia.

Our research has the following advantages. First of all, to ensure the quality of the study and reduce the influence of confounding factors, the research team will conduct a baseline assessment of the subjects. The subjective evaluation results will be obtained by using PSQI, HAMA, Generalized anxiety scale (GAD-7), and Insomnia Severity Index (ISI). The emotional changes of the subjects during the experiment will be recorded objectively by the study members (29). Through the analysis of a large number of papers, Baihui, Sanyinjiao, Shenmen, Yintang, and Shenting are recognized as acupoints with good curative effects in the treatment of insomnia (30–32). This experiment does not use skin-breaking intervention, but self-acupoint pressing intervention to ensure the safety and controllability of the trial to a certain extent, and increase the feasibility of the trial. Secondly, the subjectivity of the subjects in this experiment is strong. To ensure the validity of the experimental research data and reduce the deviation caused by the amount of operation or non-standard operation, senior acupuncture and massage lecturers and Taijiquan professional teachers are arranged to conduct regular acupoint pressing and Taijiquan training for the subjects so that the subjects can carry out the standard operation in the prescribed treatment period. In addition, most of the subjects in this experiment have a certain tendency toward anxiety and depression. To maintain the physical and mental health of the subjects and ensure that the subjects could complete the research experiment, the research team specially equipped professional psychological counselors to carry out regular psychological counseling, and the researchers recorded their emotions to prevent overreaction in the research process. In the course of the experiment, the research team will collect the subjective scale data and objective emotional records of the subjects every 3 weeks, and analyze the effectiveness and difference of Taijiquan combined with acupoint pressing therapy in treatment groups and control group (33, 34). After 12 weeks of intervention, all participants will begin an unsupervised follow-up period of another 12 weeks to restore their daily lifestyle and re-use the results of the scale to analyze the long-term effect of combined therapy. In Chinese clinical research trials, the effects of Taijiquan and acupoint pressing in the treatment of insomnia will vary with different intervention times, so the intervention time in this experiment is also the expansion angle of the combined therapy, which will also be the focus of this study report.

This study has certain limitations. The research team cannot use the blind method for the test subjects, so the test process adopts the random distribution sequence results. The test treatment period and grouping process are confidential to the subjects, and the evaluators and data statistical analysts are blinded (35). The random distribution information cannot be disclosed until the research and test are completely completed. At the same time, subjects are not allowed to communicate treatment measures with each other during the trial, so as not to increase the influence of confounding factors.

This experimental study will collect experimental data from subjective and objective to analyze the effectiveness of Taijiquan combined with acupoint pressing in the treatment of anxiety insomnia of college students. It is of great significance for clinical practice to find the best time for the treatment of anxiety insomnia in college students (36). The results of the follow-up data of subjects are also expected to evaluate the effectiveness of Taijiquan combined with acupoint pressing as adjuvant therapy or preventive intervention of specific health status in the treatment of clinical anxiety insomnia.

## Ethics statement

This experiment has been approved by the Medical Ethics Committee of the Hospital of Chengdu University of Traditional Chinese Medicine, and the ethical approval mark is 2022KL-004.

## Author contributions

XF, YC, and JYD participated in the conception and design of the trial. JYD, XL, YW, LY, and JF participated in drafting the manuscript. JMD, JX, and YY were responsible for participants recruitment. PL and ZS were in charge of data collection. WP and QH conducted data analysis. All authors discussed, read, and revised the manuscript and approved the publication of this protocol.

## Abbreviations

PSQI: Pittsburgh sleep quality index
HAMA: Hamilton Anxiety Scale
GAD-7: Generalized Anxiety Disorder
ISI: Insomnia Severity Index.
